# Metformin improves cognitive impairment in diabetic mice induced by a combination of streptozotocin and isoflurane anesthesia

**DOI:** 10.1080/21655979.2021.2004978

**Published:** 2021-12-01

**Authors:** Weiwei Zhang, Lingxia Zhao, Jianwen Zhang, Pengfei Li, Zhigan Lv

**Affiliations:** a Department of Anesthesiology, Shanxi Academy of Medical Sciences, Taiyuan, P.R. China; b Department of Anesthesiology, Tongji Hospital, Tongji Medical College, Huazhong University of Science and Technology, Wuhan, P.R. China; c Translational Medicine Research Center of Shanxi Medical University, Taiyuan, P.R. China

**Keywords:** Metformin, cognitive impairment, isoflurane, streptozotocin, diabetic mice

## Abstract

To investigate the protective effects of metformin on the diabetic mice with cognitive impairment induced by the combination of streptozotocin (STZ) and isoflurane anesthesia. The isoflurane-anesthetized cognitive impairment model mice were established and then observed via behavioral tests and histopathological examination. Then these model mice were randomly assigned to three groups, which received the PBS, low and high doses of metformin, respectively. The body weight, food and water consumption of model mice were measured every other day. The mechanisms of metformin on ameliorating the cognitive dysfunction were further investigated by histomorphological, biochemical and Western blot analysis. After 14-days treatment of metformin, the diabetic symptoms in STZ-induced diabetic mice were significantly alleviated. Metformin could restore the isoflurane- and STZ-induced hippocampal tissue damage, cognitive and memory impairment in exposed space via improving the oxidative stress, upregulating the contents of glucagon-like peptide-1 (GLP-1) and glucose-dependent insulinotropic polypeptide (GIP) in the hippocampus tissues of diabetic mice. Furthermore, chronic treatment of metformin significantly down-regulated the expression of AGEs, RAGE, pNF-κB, iNOS, and COX-2. In conclusion, metformin can improve the isoflurane- and STZ-induced cognitive impairment in diabetic mice via improving oxidative stress and inhibiting the AGEs/RAGE/NF-κB signaling pathway.

## 1. Introduction

Anesthesia is often an inevitable medical intervention in many patients undergoing surgery [1]. Patients after anesthesia are prone to postoperative cognitive dysfunction (POCD), mainly manifested as progressive memory loss, acute cognitive dysfunction or cognitive deterioration, and in severe cases, loss of independent living ability [2–4]. Recently, accumulating evidence has shown that diabetes can also induce central nervous system complications and cause cognitive dysfunction [5,6]. About 60% to 70% of diabetic patients will eventually develop cognitive dysfunction, the mechanism, however, has not been fully clarified [6]. Some studies have shown that diabetes-induced cognitive dysfunction is associated with hyperglycemia, abnormal insulin signaling pathways as well as oxidative stress [7,8]. In addition, like POCD, the deposits of microtubule-associated protein tau (Tau) and β-amyloid were occurred in the brains of diabetic patients, indicating the similar pathophysiological processes in the brains between diabetic patients and POCD patients [9,10].

Metformin is currently the most widely used oral hypoglycemic agent [11]. Metformin was shown to ameliorate type 2 diabetes (T2D) mainly by improving insulin resistance and promoting glucose uptake [11,12]. In addition, metformin has also received much attention in the field of neurodegenerative disease [13]. Recent studies have shown that Metformin can rapidly cross the blood-brain barrier after being absorbed into the blood and accumulate at high concentrations in most brain regions, thus exerting beneficial neuroprotective effects on the aging brain and cognitive functions [14]. Furthermore, a clinical study by Hsu et al showed that metformin can significantly reduce the risk of cognitive impairment in patients with T2DM [15]. Other two larger cohort studies also suggested that taking metformin and sulfonylurea hypoglycemic agents can reduce the risk of dementia in patients with T2D, with metformin reducing dementia more significantly [16,17]. Considering the chronic course of T2D patients, Guo et al. demonstrated that long-term metformin treatment significantly improved memory index and increased attention in patients with T2DM [18]. However, to our knowledge, the effect of metformin on diabetes- and anesthesia-induced cognitive impairment remains to be elucidated.

In order to confirm whether chronic treatment of metformin contributed to improve the cognitive dysfunction in anesthetized diabetic mice. The diabetic mice with cognitive impairment were induced by the combination of isoflurane and STZ. The development of cognitive impairment of diabetic mice was observed by behavioral test and histopathological examination. In addition, the underlying mechanisms of metformin on ameliorating the cognitive dysfunction induced by STZ and isoflurane were also investigated.

## 2. Materials and methods

### 2.1 Reagents and animals

Metformin (Aladdin Biotechnology, M107827, Shanghai, China); Mouse insulin (EMINS), GLP-1 (BMS2194), GIP (PA5-76867) and advanced glycation end products (AGEs, PA1-075) ELISA kit (Thermo Fisher Scientific, MA, USA); Malondialdehyde (MDA, MAK085), superoxide dismutase (SOD, CS0009), glutathione peroxidase (GSH-Px, CGP1), and catalase (CAT, CAT100) assay kit (Sigma-Aldrich, Munich, Germany); Water maze and Water Labyrinth Video Tracking Analysis System (TECHMAN Software Co. Ltd., Chengdu, China); Other reagents not otherwise specified (Sigma-Aldrich, Munich, Germany).

Male C57BL/6 J mice were obtained from Laboratory Animal Center of Huazhong University of Science and Technology. All mice were bred in the animal facility with groups of 6 per cage under the SPF condition of a standard 12-hour light/dark cycle for acclimation. All the animal experiments were performed according to guideline and approved by Laboratory Animal Center of Huazhong University of Science and Technology with approval of HUST00200415R06.

### 2.2 Establishment of isoflurane-anesthetized diabetic mice

After a week of adaptation, the mice in model and normal group were all fasted overnight, and then received administrated with single dose of STZ at the dose of 50 mg/kg and the same volume of citrate buffer, respectively. When the random blood glucose level (BGL) of mice administered with STZ for 72 h was greater than 16.7 mmol/L, it was considered that the diabetic model was successfully established. Then the diabetic mice were placed in an anesthetic chamber pre-filled with 98.5% O_2_ containing 1.5% isoflurane (1.5 L/min, 2 h) for isoflurane exposure. The temperature of the anesthesia chamber was controlled at 37 ± 0.5°C. After gas exposure, mice were put back to their home cage for emergence and supplied with 1.5 L/min of 100% O_2_. Mice in the normal group were placed in the same chamber which pre-filled with 100% O_2_ (1.5 L/min, 2 h).

Isoflurane-anesthetized diabetic mice were randomly divided into 3 groups: (1) Control group (mice received saline injection for 14 days); (2) Low-dose metformin treated group (mice received metformin intragastric administration with 50 mg/kg/day for 14 days); (3) High-dose metformin treated group (mice received metformin intragastric administration with 250 mg/kg/day for 14 days). Body weight, food and water consumption of animals were measured once every two days. Oral glucose tolerance was detected at the end of chronic treatment. In brief, after 15 h fasting, all mice models were subcutaneously administrated with 2 g/kg glucose and the blood glucose levels were detected at time point of 0, 0.25, 0.50, 0.75, 1.00 and 2.00 h after glucose loading by using the hand-held blood glucose monitor (Johnson & Johnson, America). Serum insulin levels of mice was detected by ELISA method.

### 2.3 Morris water maze test

**Figure 1.**
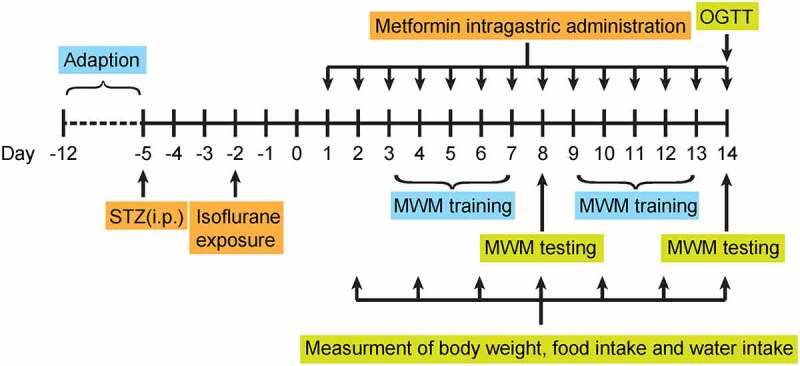
Schematic diagram of the experimental schedule

Morris water maze test was used to evaluate the learning and working memory of isoflurane-anesthetized diabetic mice as previously described [19]. The examiner was blinded to each group. Briefly, a water-filled circular pool (diameter × height: 130 × 50 cm; water depth: 31 cm, water temperature: 25.0 ± 2.0°C) was equally divided into 4 quadrants and a submerged circular platform was set at one of the quadrants. The swimming paths of mice were video-tracked by a video camera fixed to the ceiling of the room and analyzed by MT-200 Water Labyrinth Video Tracking Analysis System. As shown in Figure 1, from 3 days after successful modeling, all mice were randomly assigned to different starting points and trained to swim to the hidden platform within 120 s then allowed to stay on it for 15 seconds. If the mouse cannot find the platform within 120 s, it will be guided to the platform and allowed to stay on it for 15 s. Escape latency was recorded as the time for each mice reaching the platform. Training was performed four times a day with 60 seconds rest after each session. On day 5, the previous training was repeated and then the platform was removed. Mice were randomly assigned to different starting points and swam for 120 s. The software recorded the number of original platform position crossing as well as the time spent in the target quadrant of each mouse.

One day after the probe trial was completed, the platform was moved to the opposite quadrant, and all mice were undergone the same training for four times a day for 4 consecutive days. Swimming distance to find the platform and escape latency of mouse were calculated. In the transfer probe trial, the previous training was repeated and then the platform was removed. Then the mice were randomly assigned to different starting points and swam for 120 s. The software recorded the number of original platform position crossing as well as the time spent in the target quadrant of each mouse.

### 2.4 Histomorphological analysis

The histomorphological analysis of hippocampus was conducted by hematoxylin-eosin (H&E) staining. In brief, at the end of the treatment, all mice were euthanized and the brain tissue was rapidly removed and impregnated in 10% modified formaldehyde for 48 h. Subsequently, the dehydrated brain tissues were embedded in paraffin and cut into sections for 4 μm thick and baked for 20 min. Finally, H&E staining were applied for further light microscope observation. The average number of total neuron cells /field × 400 of view was used for statistical analysis.

### 2.5 Biochemical analysis

Serum insulin levels were quantified via insulin ELISA kit. At the end of treatment, hippocampal tissues of mice were collected, and ELISA method were used for the detection of MDA and AGEs concentrations, and GSH-Px, SOD and CAT activities in hippocampal tissues according to the manufacturer’s instructions.

### 2.6 Western blotting measurement

The supernatant of tissular lysate in hippocampus were prepared by 30-min cell lysis with ice-cold RIPA buffer and 15-min cryogenic centrifugation (12,000 rpm). Subsequently, the extractions of nuclear or cytoplasmic proteins in supernatant were separately performed using a nuclear or cytoplasmic extraction kit. All protein samples were prepared by SDS-PAGE, PVDF membranes transference, 5% skim milk blocking and incubation of primary antibody (antibody: diluent = 1:500) and horseradish peroxidase-labeled goat anti-rabbit secondary antibody (antibody: diluent = 1:3000). Subsequently, immunoreactive protein bands were captured with a Tanon 5200 imaging analysis system (Tanon, Shanghai, China). α-tubulin and histone-H3 were used as housekeeping proteins for cytoplasmic and nuclear lysates, respectively. Western blot bands were quantified with Image J software (V1.52a).

### 2.7 Data analysis

All data were presented as Mean ± SD. Statistical analysis was applied via GraphPad Prism 8.4 (USA) using one-way ANOVA and P values lower than 0.05 were considered as significant.

## 3. Results

In this study, we aim to confirm whether chronic treatment of metformin contributed to improve the cognitive dysfunction in anesthetized diabetic mice. The diabetic mice with cognitive impairment were induced by the combination of isoflurane and STZ. The development of cognitive impairment of diabetic mice was observed by behavioral test and histopathological examination. In addition, the underlying mechanisms of metformin on ameliorating the cognitive dysfunction induced by STZ and isoflurane were also investigated by Western blot.

### 3.1 Effects of 14-days metformin treatment on diabetes-related indicators of isoflurane-anesthetized diabetic mice

**Figure 2.**
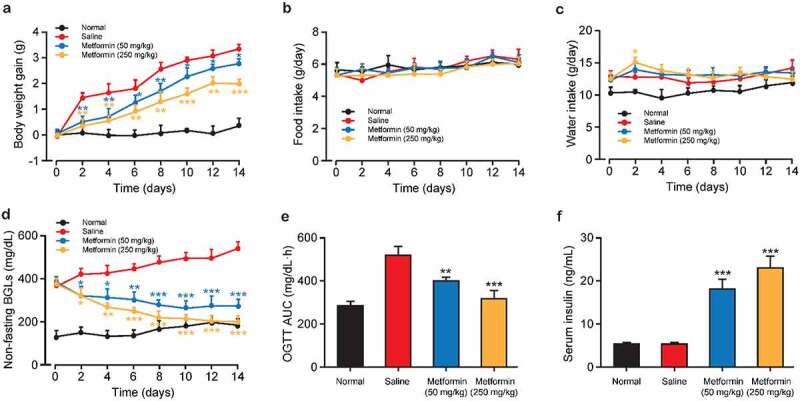
Effects of 14-day metformin administration on (a) body weight gain, average (b) daily food and (c) water consumption, (d) non-fasting BGLs, (e) OGTT AUC (0–2 h), and (f) serum insulin levels of diabetic mice. (n = 8). *p < 0.05, **p < 0.01, ***p < 0.001 vs. saline-treated group

The schematic diagram of the experimental design is shown in Figure 1. The body weight gains of mice received metformin at both two doses were notably less than that of the saline-treated group during the 2-week treatment period. However, no significant difference in body weight gain was observed between these two groups at the second week of dosing. In addition, metformin treatment did not affect food intake compared with the saline-treated group throughout the treatment period and markedly increased water intake only on day 2 (Figure 2b-c). The effect of metformin on BGLs and insulin levels in diabetic mice were also determined. Metformin treatment induced a significant reduction on non-fasting BGL in diabetic mice compared to controls on day 2 of metformin intervention and remained throughout the treatment period (Figure 2d). The results of oral glucose tolerance test showed in Figure 2e demonstrated that metformin improves glucose tolerance in diabetic mice to almost similar levels to normal mice. Moreover, serum insulin levels were obviously higher in both diabetic mice received both two doses of metformin relative to that of the saline-treated ones at the end of treatment (*p* < 0.01 and *p* < 0.001 for 50 and 250 mg/kg, respectively, Figure 2e). Above results collectively showed that metformin significantly alleviated the diabetic symptoms in STZ-induced diabetic mice throughout the 14-day treatment.

### 3.2 Effect of metformin treatment on spatial learning and memory of diabetic mice with cognitive dysfunctions

**Figure 3.**
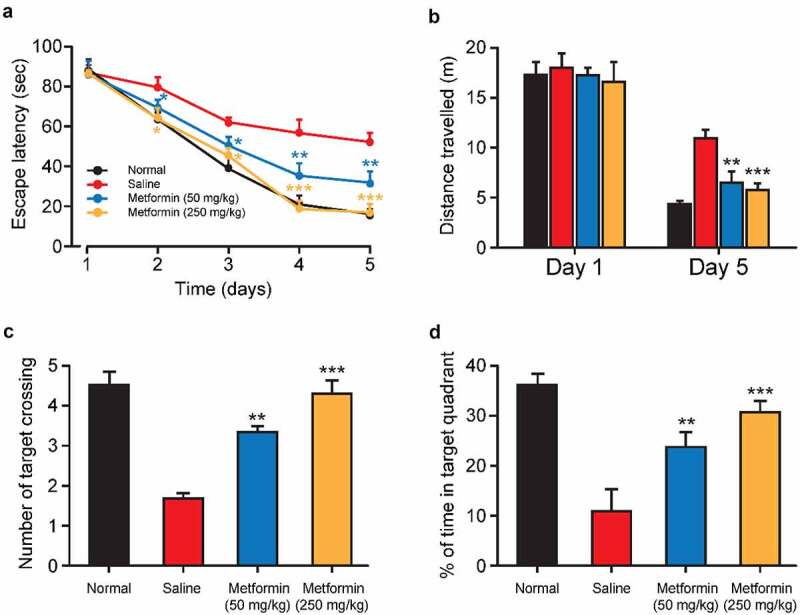
Effect of metformin treatment on spatial learning and memory of mice with isoflurane- and STZ-induced cognitive dysfunctions. (a) Escape latency, (b) swimming distance, (c) the mean number of platform location crossing, and (d) the percentage of time spent in the target quadrant of mice with isoflurane- and STZ-induced cognitive dysfunctions (n = 8). **p < 0.01, ***p < 0.001 vs. saline-treated group

To further assess the beneficial effects of metformin on isoflurane- and STZ-induced cognitive dysfunction, the spatial learning and memory of isoflurane-anesthetized diabetic mice were evaluated via Morris water maze test. As the results showed in Figure 3a, the gradually decreased escape latency for each group during the five days of training. Interestingly, Metformin treated group exhibited the significantly shorter escape latency on the third to fifth days of training trials compared to the saline-treated group, indicating a significant improvement of spatial learning function. Moreover, the traveled distances of mice searching for an underwater platform were also recorded (Figure 3b). There was no significant difference in the moving distance for searching hidden platform among all groups on the first training day. Nevertheless, the traveled distance in the saline-treated group was clearly increased on the fifth training day relative to that in the normal group, whereas metformin treatment significantly reversed these changes in a dose-dependent manner. On the sixth day, the probe trial was performed in the platform-removal pool. The mean number of passes at the platform position and the percentage of time spent in the target quadrant are shown in 2 C – D. Significant decrease in the mean number of platform-crossing and the percentage of time spent in the target quadrant demonstrated obvious memory impairment induced by isoflurane and STZ in control group. Nevertheless, chronic treatment of metformin at both two doses notably enhanced the frequencies of the target quadrant crossing and prolonged the duration in the target quadrant. Taken together, metformin could restore the isoflurane and STZ induced cognitive and memory impairment.

### 3.3 Effects of metformin treatment on the working memory of mice with isoflurane- and STZ-induced cognitive dysfunctions

**Figure 4.**
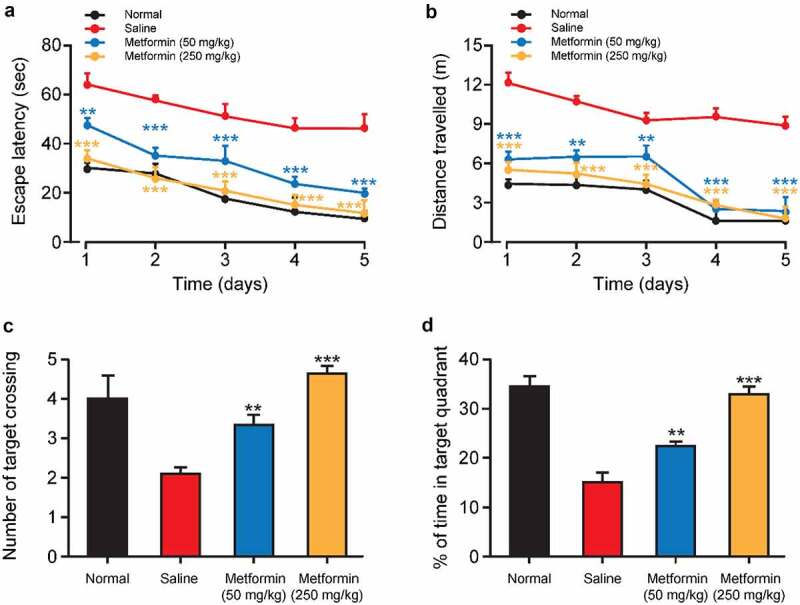
Effects of metformin treatment on spatial learning and memory of mice with isoflurane- and STZ-induced cognitive dysfunctions. (a) Escape latency, (b) swimming distance, (c) the mean number of platform location crossing, and (d) percentage of time spent in the target quadrant of mice with isoflurane- and STZ-induced cognitive dysfunctions (n = 8). *p < 0.05, **p < 0.01, ***p < 0.001 vs. saline-treated group

After completing the last probe trial, the protective effects of metformin treatment on the working memory of isoflurane-anesthetized diabetic mice were further investigated by water maze test with transfer platform. Both escape latency and swimming distance were significantly increased in the saline-treated group compared to normal mice during the training trials, implying impaired working memory induced by isoflurane and STZ (Figure 4a-b). As we expected, both the escape latency and swimming distance of metformin-treated mice were significantly shorter than those of saline-treated group. Notably, no significant difference was observed in escape latency and in swimming distance between normal group and high-dose metformin treated group. Additionally, after removing the platform, the number of times crossing the target quadrant and duration in the target quadrant were significantly reduced in the saline group (Figure 4c-d). On the contrary, metformin treatment significantly increased the number of target quadrant crossings and duration in the target quadrant. Together, these results suggested that metformin clearly improved isoflurane- and STZ-induced impairment of working memory in exposed space.

### 3.4 Effects of metformin treatment on the morphological changes in hippocampus tissues from model mice

**Figure 5.**
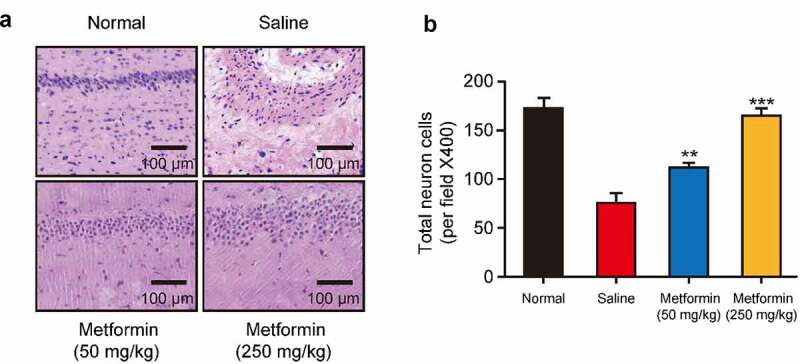
Effects of metformin treatment on histological changes of hippocampal tissues. (a) H&E staining of hippocampal tissues and (b) quantitative analysis of hippocampal neurons in mice with isoflurane- and STZ-induced cognitive dysfunctions (n = 8). *p < 0.05, **p < 0.01, ***p < 0.001 vs. saline-treated group

Neuronal damage in hippocampal tissues are the pathological basis of cognitive dysfunctions [20]. In present study, the morphological structure of hippocampal tissue was investigated by H&E staining. As shown in Figure 5a, the hippocampal tissues of normal mice showed no obvious neuronal abnormalities, and CA1 pyramidal cells were arranged neatly and tightly with intact structure. However, the hippocampal tissue from saline-treated model group showed significant disintegration of the pyramidal lamellar structure and fixed contraction of neuronal nuclei with enlargement and disarrangement of the extracellular space. Chronic treatment of metformin at both doses significantly attenuated these abnormalities. Furthermore, in both doses of metformin treated group, the neurons in the hippocampus showed better cell morphology, neatly arrangement and homogeneous staining. Particularly, the hippocampal tissue in high-dose metformin treated group showed a greater number of hippocampal neuronal cells, and further quantitative analysis of neuronal cells showed that metformin treatment significantly increased the number of neuronal cells in hippocampal tissue (Figure 5b), indicating that metformin significantly ameliorated isoflurane- and STZ-induced hippocampal tissue damage in diabetic mice with cognitive dysfunction.

**Figure 6.**
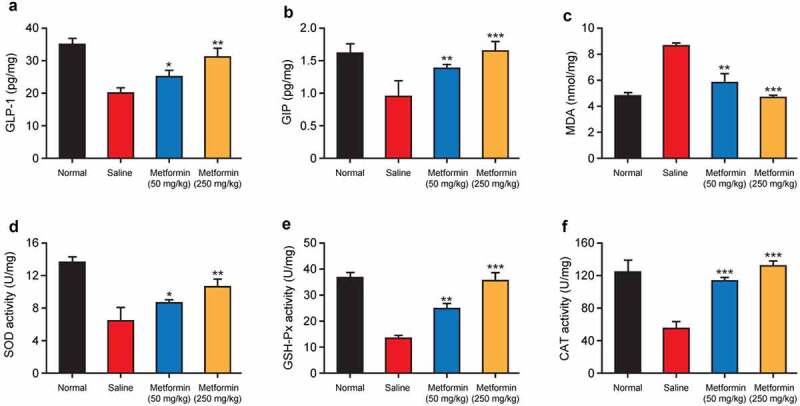
Effects of metformin treatment on incretin concentration and oxidative stress of hippocampal tissues from model mice. The levels of (a) GLP-1, (b) GIP, and (c) MDA and the activities of (d) SOD, (e) GSH-Px, and (f) CAT in hippocampal tissues of mice with isoflurane- and STZ-induced cognitive dysfunctions (n = 8). *p < 0.05, **p < 0.01, ***p < 0.001 vs. saline-treated group

### 3.5 Effect of metformin treatment on the incretin concentration and oxidative stress in hippocampal tissues

GLP-1 and GIP receptors are widely distributed in the hippocampus, and both GLP-1 and GIP are closely associated with synaptic plasticity controlling and memory formation [21]. Recently, GLP-1 and GIP analogues have been reported to inhibit the memory impairment and cognitive dysfunction in neurodegenerative diseases [22]. Therefore, the contents of GLP-1 and GIP in hippocampal tissue homogenates were determined. As showed in Figure 6a-b, the contents of GLP-1 and GIP in hippocampus in saline-treated group were remarkably lower than those in the normal mice (both *p* < 0.01). Nevertheless, GLP-1 and GIP contents in hippocampal tissues were significantly increased after chronic treatment of metformin at both two doses (both *p* < 0.05).

Oxidative stress is deeply linked to the pathology of cognitive dysfunctions. The results in Figure 6c-f revealed that low-dose metformin intervention protects the hippocampal tissues from oxidative stress, manifested as enhanced activity of vital antioxidant enzymes, including SOD, GSH-Px, and CAT as well as a reduction of neurotoxic major end-products of lipid peroxidation, MDA. In addition, these beneficial effects were further enhanced by high-dose metformin treatment, suggesting that metformin can ameliorate isoflurane- and STZ-induced oxidative stress in hippocampal tissues of diabetic mice.

### 3.6 Effects of metformin treatment on expression of key regulators involved in the RAGE/NF-κB signaling pathway

**Figure 7.**
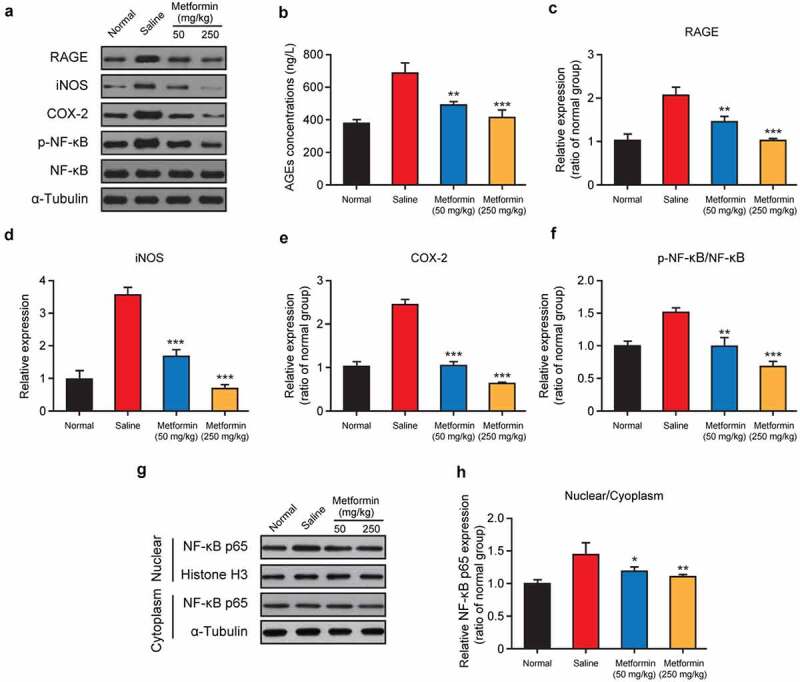
Effect of metformin treatment on expression of key regulators involved in the RAGE/NF-κB signaling pathway. (a, g) Western blot analysis, (b) AGEs concentration, and relative expression levels of (c) RAGE, (d) iNOS, (e) COX-2, (f) the ratio of phosphorylated NF-κB and NF-κB, and (h) quantitative analysis of NF-κB p65 nuclear translocation in hippocampal tissue of mice with isoflurane and STZ induced cognitive dysfunction (n = 8). *p < 0.05, **p < 0.01, ***p < 0.001 vs. saline-treated group

AGEs, as a series of major factors, were involved in pathological development of diabetes- or anesthesia-induced cognitive dysfunctions [23]. To further elucidate the regulatory mechanism of metformin on cognitive impairment induced by isoflurane and STZ, the key regulators involved in NF-κB signaling pathway were further analyzed. Firstly, the content of AGEs in mouse hippocampus were measured by ELISA method, and the results showed in Figure 7b demonstrated that the AGEs content in the saline-treated group was remarkably increased relative to the normal group (*p* < 0.01). Especially the metformin intervention at both doses significantly decreased the AGEs content (both *p* < 0.01). Similarly, Western blot analysis exhibited a dramatic increase in the expression of AGEs receptor (RAGE) in the model group, but these changes were significantly reversed by metformin treatment (50 mg/kg/day and 250 mg/kg/day, Figure 7a-c). As evidenced by obviously higher expression levels of two kinds of critical inflammatory factors involved in the NF-κB signaling pathway, iNOS and COX-2, the binding of AGEs to RAGE ultimately resulted in the inflammatory factors generation and the activation of NF-κB signaling pathway. In present study, chronic treatment of metformin could significantly down-regulate the expression of these two factors (both *p* < 0.001, Figure 7d-e). Moreover, 2-week treatment of metformin at both two doses significantly reduced the expression level of phosphorylated NF-κB compared to the saline-treated group (both *p* < 0.01), while also effectively blocked the translocation of cytoplasmic NF-κB p65 into nucleus (figure 7f-h).

## 4. Discussion

Diabetes and anesthesia are major risk factors contributing to cognitive dysfunctions, such as vascular dementia and Alzheimer’s disease [24]. Although strict glycemic control could reduce cognitive impairment in diabetes, the benefits of anti-diabetic agents in preventing cognitive dysfunction induced by the combination of diabetes and anesthesia has not been established [25,26]. Metformin is recommended as a base medication for all T2D patients without contraindications [12]. To our knowledge, metformin can also decelerate the progression of neurodegenerative diseases and has a positive effect on the improvement of cognitive dysfunction in T2D patients [13,14]. Previous studies have shown that STZ-induced diabetic mice are characterized by impaired cognitive performance so that being considered as a useful model to explore cognitive impairment attributable to diabetes [8]. Therefore, we investigated the effect of metformin treatment on cognitive function in isoflurane-anesthetized diabetic mice. Consistent with the results reported in the previous literatures [27], 2-week metformin treatment significantly reduced body weight and BGL in diabetic mice (Figure 2). Furthermore, oral glucose tolerance was also significantly improved in diabetic mice after the end of treatment, accompanied by an increase in serum insulin levels. These results indicate that metformin treatment significantly ameliorates diabetic symptoms in isoflurane-anesthetized diabetic mice.

Morris water maze test is one of the most particularly useful tools for behavioral assessment of rodents [28]. In present study, the hidden platform acquisition test and the probe test were performed to evaluate the spatial learning and spatial memory of mice with isoflurane and STZ induced cognitive and memory impairment. As the result showed in Figure 3, isoflurane-anesthetized and STZ-induced cognitively impaired mice showed notable increase in escape latency and traveled distance over the 5 days of training. In addition, the mean number of times that mouse crossing the platform location as well as the percent time that mouse spent in the target quadrant were also significantly reduced in the model group mice in the exploratory experiment (Figure 4). These results collectively demonstrated the STZ- and isoflurane-induced spatial learning and memory impairment. Importantly, once-daily metformin treatment significantly reduced escape latency and traveled distance in diabetic mice, and significantly increased the percent time that mouse spent in the target quadrant and the number of platform crossing (Figure 3). Subsequently, we tested the working memory function of mice in a water maze with a transfer platform and the results indicated that mice with isoflurane and STZ-induced cognitive impairment were hardly to forming good working memory for the new platform location (Figure 4). Significantly, the metformin-treated group showed lower escape latency and swimming distance, as well as more number of times that mouse crossing the platform location and prolonged duration in the target quadrant compared with the saline-treated group. Hence, we are confident that the metformin could significantly improve cognitive impairment induced by isoflurane and STZ.

The hippocampus is a key structure for cognition, particularly the functions of learning and memory [29]. Several studies have shown that neuronal loss in hippocampal tissue is a key factor leading to impaired cognitive function [30,31]. Therefore, the effects of metformin on isoflurane- and STZ-induced hippocampal tissue damage were investigated. As the results showed in Figure 5, metformin significantly ameliorated the pathological lesions in hippocampal tissues, as evidenced by intact, neatly arranged, and homogeneously stained hippocampal neuronal structures in mice treated with metformin. Moreover, 2-week metformin treatment also brought the significantly increased number of neurons in the hippocampal tissue. Incretin levels and the degree of oxidative stress are closely related to the pathology of cognitive dysfunction. Moreover, the metformin treatment significantly increased GLP-1 and GIP levels and enhanced SOD activity in hippocampal tissues, while decreased the MDA levels (Figure 6). Above data collectively supported the benefits of metformin on isoflurane anesthesia and STZ-induced cognitive impairment in mice.

Neuroinflammatory mechanisms are closely related to the development of cognitive dysfunction induced by anesthesia or diabetes [32]. In particular, AGEs is deeply linked to the inflammatory response during the development of cognitive dysfunction [23]. AGEs are widely distributed and are mainly found in the plasma, hippocampus, and brain of diabetic patients [23]. Therefore, many studies suggested that AGEs may be involved in anesthesia- or diabetes-induced cerebral neuroinflammation, which leads to cognitive dysfunction. Meanwhile, AGEs binding to RAGE could trigger the activation of NF-κB signaling pathways, phosphorylate the NF-κB, and induce the expression of inflammatory factors, such as iNOS and COX-2, thereby mediating cognitive dysfunction caused by neuroinflammation. Based on this, the effect of metformin on RAGE/NF-κB signaling pathway was investigated, and the results were shown in Figure 7. Two-week metformin treatment significantly down-regulated the expression of AGEs, RAGE, phosphorylated NF-κB, iNOS, and COX-2, indicating that metformin ameliorated isoflurane- and STZ-induced cognitive impairment by inhibiting the AGEs/RAGE/NF-κB signaling pathway.

The current study has several limitations. First, we assessed the effect of metformin on cognitive dysfunction induced by a combination of anesthesia and diabetes in mice. However, the effects of anesthesia and diabetes could be different on cognitive function in mice. The results of the current study have established a system for investigating the effects of different drugs on anesthesia- and/or diabetes-induced cognitive dysfunction. Second, our current study cannot determine whether other signaling pathways are involved in the development of neuroinflammation in mouse hippocampal tissue. In addition to the AGEs/RAGE signaling pathway, whether there are other signaling pathways involved in the phosphorylation of NF-κB and how this affects cognitive function in mice warrants further investigation. Finally, there are also some studies showing that metformin aggravates cognitive dysfunction [33,34]. We did not address the effect of metformin treatment on cognitive function in normal mice throughout the experiment. We will supplement this portion of the data in future studies.

## 5. Conclusion

In summary, this present study firstly demonstrated that metformin can notably improve the diabetes-associated symptoms, histomorphology changes of hippocampal tissue, and cognitive impairment induced by isoflurane anesthesia and STZ in mice, thus highlighting metformin as a potentially promising agent to prevent anesthesia- and diabetes-induced cognitive impairment. Apart from this, further in-depth exploration will be needed to delineate the underlying mechanisms of metformin ameliorating diabetes- and anesthesia-induced cognitive impairment.

## Data Availability

All data generated or analyzed during this study are included in this article.

## Highlight

- Metformin improved spatial learning and working memory of diabetic mice
- Metformin ameliorated neuronal damage in hippocampal tissue of diabetic mice
- Metformin suppressed oxidative stress of isoflurane-anesthetized diabetic mice
- Metformin ameliorated cognitive impairment via inhibiting AGEs/RAGE/NF-κB pathway
